# Vascular Health Is Associated With Functional Connectivity Decline in Higher-Order Networks of Older Adults

**DOI:** 10.3389/fnint.2022.847824

**Published:** 2022-04-26

**Authors:** Miranka Wirth, Malo Gaubert, Theresa Köbe, Antoine Garnier-Crussard, Catharina Lange, Julie Gonneaud, Robin de Flores, Brigitte Landeau, Vincent de la Sayette, Gaël Chételat

**Affiliations:** ^1^German Center for Neurodegenerative Diseases (DZNE), Dresden, Germany; ^2^Clinical and Research Memory Center of Lyon, Lyon Institute for Aging, Hospices Civils de Lyon, Lyon, France; ^3^INSERM 1048, CNRS 5292, Neuroscience Research Centre, Lyon, France; ^4^UNICAEN, INSERM, U1237, PhIND “Physiopathology and Imaging of Neurological Disorders,” Institut Blood and Brain @ Caen-Normandie, Cyceron, Normandy University, Caen, France; ^5^Department of Nuclear Medicine, Charité – Universitätsmedizin Berlin, Corporate Member of Freie Universität Berlin and Humboldt-Universität zu Berlin, Berlin, Germany; ^6^Department of Neurology, CHU de Caen, Caen, France

**Keywords:** aging, longitudinal resting-state functional connectivity, cognition, vascular risk, modifiable risk factors

## Abstract

**Background:**

Poor vascular health may impede brain functioning in older adults, thus possibly increasing the risk of cognitive decline and Alzheimer’s disease (AD). The emerging link between vascular risk factors (VRF) and longitudinal decline in resting-state functional connectivity (RSFC) within functional brain networks needs replication and further research in independent cohorts.

**Method:**

We examined 95 non-demented older adults using the IMAP+ cohort (Caen, France). VRF were assessed at baseline through systolic and diastolic blood pressure, body-mass-index, and glycated hemoglobin (HbA1c) levels. Brain pathological burden was measured using white matter hyperintensity (WMH) volumes, derived from FLAIR images, and cortical β-Amyloid (Aβ) deposition, derived from florbetapir-PET imaging. RSFC was estimated from functional MRI scans within canonical brain networks at baseline and up to 3 years of follow-up. Linear mixed-effects models evaluated the independent predictive value of VRF on longitudinal changes in network-specific and global RSFC as well as a potential association between these RSFC changes and cognitive decline.

**Results:**

We replicate that RSFC increased over time in global RSFC and in the default-mode, salience/ventral-attention and fronto-parietal networks. In contrast, higher diastolic blood pressure levels were independently associated with a decrease of RSFC over time in the default-mode, salience/ventral-attention, and fronto-parietal networks. Moreover, higher HbA1c levels were independently associated with a reduction of the observed RSFC increase over time in the salience/ventral-attention network. Both of these associations were independent of brain pathology related to Aβ load and WMH volumes. The VRF-related changes in RSFC over time were not significantly associated with longitudinal changes in cognitive performance.

**Conclusion:**

Our longitudinal findings corroborate that VRF promote RSFC alterations over time within higher-order brain networks, irrespective of pathological brain burden. Altered RSFC in large-scale cognitive networks may eventually increase the vulnerability to aging and AD.

## Introduction

The prevention and early treatment of modifiable risk factors for Alzheimer’s disease (AD) is of utmost importance to preserve brain health and well-being in the aging population (Livingston et al., 2020). Besides neuropathological hallmarks of β-Amyloid (Aβ) and tau, AD is characterized by early functional alterations in intrinsic brain networks (Iturria-Medina et al., 2016). Functional connectivity within canonical networks can be assessed at resting-state, using fluctuations in the blood oxygen level dependent (BOLD) signal of functional magnetic resonance imaging (MRI) as an indirect measure of neuronal activity (Fox and Raichle, 2007). While resting-state functional connectivity (RSFC) changes are proposed to convey diagnostically meaningful information in at-risk stages of AD (Vemuri et al., 2012), the identification of potentially modifiable instigators underlying these RSFC alterations is an ongoing research topic.

There is evidence to suggest that poor vascular health could be a driving factor of functional brain alterations in healthy and pathological aging. Vascular risk factors (VRF) including hypertension, obesity and diabetes are commonly found in the older adults (Virani et al., 2021), where they may increase the risk of incident cognitive impairment and AD dementia (Kivipelto et al., 2001; Luchsinger et al., 2005; Schrijvers et al., 2010). Existing, mostly cross-sectional studies have shown that VRF, such as elevated levels of cholesterol, blood pressure, or body mass index (BMI), are related to altered RSFC within large-scale brain networks. Those comprise higher-order (cognitive) networks, mainly the default-mode (DMN), salience (SAL), or the fronto-parietal (FPN) networks (Kullmann et al., 2012; Li et al., 2015; Zhang et al., 2016), which are also robustly affected by aging and AD (Buckner et al., 2009; Elman et al., 2016; Grady et al., 2016; Badhwar et al., 2017). Given that even subtle brain alterations as imposed by VRF may constitute early events in the pathogenic cascade of AD (Zlokovic, 2011), vascular health may serve as promising modifiable target for early intervention (de la Torre, 2010).

To overcome the limitation of cross-sectional studies regarding causal interpretations, longitudinal research works are necessary. Therefore, an author of the present study and colleagues (Köbe et al., 2021) recently examined longitudinal data from the PREVENT-AD cohort (Breitner et al., 2016; Köbe et al., 2020). In older adults with a family history of sporadic AD, the authors found an overall increase of RSFC over time across the entire sample. This longitudinal increase was seen in global RSFC (i.e., averaged connectivity of each gray matter region with every other gray matter region) and in network-specific RSFC including the limbic and dorsal attention networks. In contrast, baseline VRF were independently associated with a reduction of RSFC over time (Köbe et al., 2021). Namely, higher diastolic blood pressure levels and higher blood cholesterol levels (both total and low density lipoprotein) independently predicted a decrease of RSFC over time comprising (but not limited to) the DMN. Moreover, these associations were found irrespective of concomitant Aβ deposition and there was no measurable impact of Aβ on changes in RSFC over time.

Overall, these findings suggest a critical role of modifiable VRF in the emergence of RSFC alterations within higher-order brain networks in older adults. Yet, supporting evidence for this observation from independent cohorts is needed. In the current study, we essentially aimed to replicate and extend the existing longitudinal findings (Köbe et al., 2021). We therefore assessed the impact of VRF on RSFC trajectories over time in non-demented older adults using an independent cohort, namely the IMAP+ cohort. This cohort features longitudinal assessments of neurocognitive, neuropsychological, and neuroimaging measures (e.g., Mutlu et al., 2017; Gaubert et al., 2021) similar to the PREVENT-AD cohort. We evaluated VRF related to systolic and diastolic blood pressure, BMI, and glycated hemoglobin (HbA1c), a long-term marker of glucose metabolism. Our main objective was to examine the independent impact of the VRF on RSFC trajectories within established large-scale brain networks, as done previously (Köbe et al., 2021). In accordance with the previous findings, we hypothesized that higher levels of selective VRF would be predictive of changes in RSFC over time, primarily within higher-order brain networks that are susceptible to aging and AD.

In addition, we incorporated some novel aspects in our study to advance this field of research: Here, we assessed the effect of HbA1c levels on RSFC trajectories and accounted for brain pathology of Aβ deposition and white matter hyperintensities (WMH), a sensitive neuroimaging marker of cerebral small vessel disease (Prins and Scheltens, 2015). We further explored a potential cognitive relevance of the VRF-related changes in RSFC over time, given that poor vascular health may accelerate cognitive decline in older adults (Jefferson et al., 2015; Rabin et al., 2018; Yaffe et al., 2021).

## Materials and Methods

### Participants and Study Design

**FIGURE 1:**
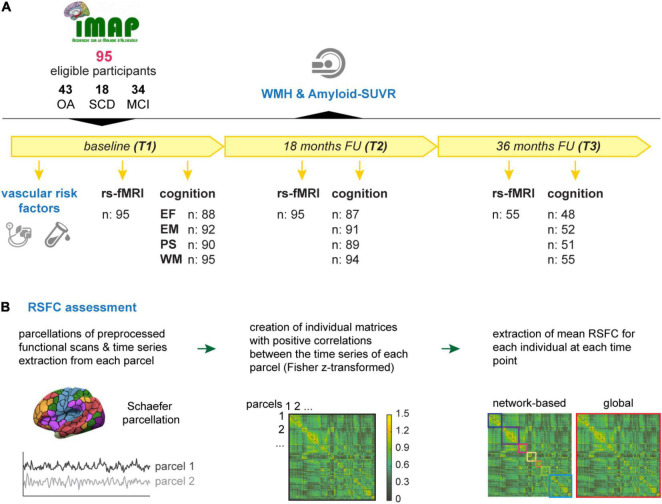
Overview on the study design and RSFC assessment. **(A)** In total, 95 eligible participants of the IMAP+ cohort were followed over up to 3 years with at least two and maximal three timepoints (T1 – T3). The vascular risk factors (blood pressure, BMI, and HbA1c) were assessed at baseline. The earliest available measurement of white matter hyperintensities (WMH) and Aβ-PET SUVR, that were acquired along the course of the study, were considered in the current analyses. Resting-state functional magnetic resonance images (rs-fMRI) were acquired at T1 and T2 for all participant, 55 of whom also had a valid scan at T3. Cognitive performance was operationalized by composite scores of executive function (EF), episodic memory (EM), processing speed (PS), and working memory (WM) and was assessed at T1, T2, and T3. Composite scores could not be calculated for some participants due to missing data; valid numbers are shown in the figure. **(B)** The assessment of RSFC was carried out as follows: Individual time series of pre-processed scans were extracted from each parcel of the Schaefer parcellations atlas (Schaefer et al., 2018). Correlation matrices were obtained by correlating the time series of each parcel with one another. Correlation matrices were Fisher z-transformed and thresholded by only keeping positive correlations. Extracted mean correlations represent the indirect measure of network-based (multicolored squares) and global (red square) RSFC. BMI, body-mass-index; FU, follow up; HbA1c, glycated hemoglobin A1; MCI, mild cognitive impairment; RSFC, resting-state functional connectivity; SCD, subjective cognitive decline; Amyloid-SUVR, florbetapir standard uptake value ratio; OA, older adults.

All participants were part of the IMAP+ cohort (“Imagerie Multimodale de la Maladie d’Alzheimer à un stade Précoce,” Caen, France, ClinicalTrials.gov: NCT01638949), a multimodal neuroimaging study of early Alzheimer’s disease. In the longitudinal study protocol, over up to three timepoints (baseline/T1; 18-months follow-up/T2; and 36-months follow-up/T3) VRF, MRI, PET imaging and neuropsychology are assessed in close temporal proximity (within 3 months). Given the longitudinal and multifactorial approach of the current analyses, we selected 95 IMAP+ participants, who had at least two valid structural and functional MRI sessions to assess changes in functional connectivity over time and at least one assessment of each VRF measure of interest, WMH and florbetapir-PET across the study duration. Figure 1 provides an overview of the current study.

The present study sample comprised 43 cognitively unimpaired older adults (OA), 18 participants with subjective cognitive decline (SCD) and 34 participates with mild cognitive impairment (MCI). By including participants along the cognitive continuum with normal cognition, SCD, and MCI, we ensured that there was enough variance due to VRF and brain pathology, including of Aβ and WMH, in our sample (Wirth et al., 2018; Gaubert et al., 2021) to assess the proposed associations. Other important reasons to include the asymptomatic or preclinical stages (i.e., SCD and MCI) are that these are clinically relevant in terms of early treatment and some mechanisms may specifically appear in stages before the onset of clinical symptoms.

All IMAP+ participants were French-speaking, educated for >7 years, right-handed, aged between 51 and 85 years and had no history or clinical evidence of major neurological or psychiatric disorder, alcoholism, drug abuse, or head trauma. Uncontrolled diabetes mellitus was leading to exclusion. Participants underwent a clinical and neuropsychological examination, assessing individual’s health and multiple domains of cognition (see further details below) by a multidisciplinary team of senior neurologists and neuropsychologists. Based on this evaluation and according to internationally agreed criteria, the inclusion and group classification of the participants were conducted (McKhann et al., 1984; André et al., 2019).

The OA were recruited from the community and had cognitive performance in the normal range (i.e., within –1.65 standard deviation [SD] of the normal mean) given their age, sex, and education level in all screening tests. Participants with SCD and MCI were recruited from the local memory clinics with self-reported cognitive concerns, which were not linked to current medication, psychiatric or neurological diseases (including anxiety or depression), or other medical conditions. The patient’s independence in daily life needed to be preserved and they should not meet NINCDS-ADRDA criteria for probable AD (McKhann et al., 1984). The SCD patients reported subjective cognitive concerns in the clinical interview and in a cognitive complaint questionnaire (McNair and Kahn, 1983), in the absence of objective cognitive impairment on the neuropsychological screening as described previously (Perrotin et al., 2017; Kuhn et al., 2019). The MCI patients showed objective cognitive deficits, predominantly in episodic memory, fulfilling the clinical criteria for single or multiple domain amnestic MCI (Petersen and Morris, 2005).

The IMAP+ study was approved by the local ethics committee (Comité de Protection des Personnes Nord-Ouest III) and all participants received detailed study instructions and gave written informed consent prior to study participation.

### Assessment of Vascular Risk Factors

We assessed VRF of blood pressure, body-mass-index (BMI) and levels of hemoglobin A1c (HbA1c, long-term glucose marker). Systolic and diastolic blood pressure were measured three times consecutively at MRI or/and PET visit in a seated position. In the first and latter case, blood pressure measures were averaged over 3 (*n* = 27) or 6 (*n* = 68) assessments, respectively. Body mass index (BMI) was calculated as weight in kilograms divided by height in meters squared (kg/m^2^). The HbA1c level were measured after participants underwent a fasting blood draw. The measures were reported in our previous studies (Garnier-Crussard et al., 2020, 2021) and corresponds to the 3-month averaged blood sugar level and is related to diabetes mellitus risk. Vascular risk factors were all assessed at baseline.

### Magnetic Resonance Imaging Acquisition

Participants underwent a MRI up to three times across the study duration (T1-T3) on a 3T Achieva scanner (Philips, Netherlands) at the Cyceron Center (Caen, France). Structural high-resolution T1-weighted images were acquired first using a 3D fast field echo (FFE) sequence [3D-T1-FFE sagittal; 180 slices, no gap, slice thickness = 1 mm, in-plane resolution = 1 × 1 mm^2^, field of view = 256 × 256 mm^2^, matrix = 256 × 256, repetition time (TR) = 20 ms, echo time (TE) = 4.6 ms, flip angle = 20°]. High-resolution T2-weighted FLAIR images were obtained subsequently (3D-IR sagittal sequence, 90 slices, no gap, slice thickness = 2 mm, in-plane resolution = 0.78 × 0.78 mm^2^, TR = 8000 ms, TE = 348 ms, flip angle = 90°, field of view = 250 × 250 mm^2^, matrix = 320 × 320).

Resting-state functional MRI (rs-fMRI) scans were obtained using an interleaved 2D T2* SENSE EPI sequence designed to reduce geometric distortions using parallel imaging, short echo time, and small voxels (2D-T2*- FFE-EPI axial, SENSE = 2; 42 slices, no gap, slice thickness = 2.8 mm, in-plane resolution = 2.8 × 2.8 mm^2^, field of view = 224 × 224 mm^2^, 280 volumes, TR = 2382 ms, TE = 30 ms, flip angle = 80°, acquisition time = 11.5 min). The participants were asked to keep their eyes closed without falling asleep and to remain as still as possible during scanning. Further, participants were equipped with earplugs, their head motion was minimized by stabilizing foam pads and the scanner room was darkened. Using a subsequent debriefing questionnaire, we assured that the participants has no difficulty staying awake throughout the rs-fMRI scan and that nothing particular had disturbed their attention during the scanning.

### Magnetic Resonance Imaging Analyses

#### Structural Magnetic Resonance Imaging

The T1-weighted images were segmented, spatially normalized to the Montreal Neurological Institute (MNI) template and modulated using the standard ‘‘Segment’’ pipeline of Statistical Parametric Mapping 12 (SPM12^1^, release 7487, Wellcome Department of Cognitive Neurology, London, United Kingdom; MATLAB R2018a; MathWorks, Natick, MA, United States). Total intracranial volume (TIV) was estimated from the subsequently segmented gray matter, white matter and cerebral spinal fluid volumes, using the “Tissue volume” routine of SPM12.

#### Assessment of White Matter Hyperintensity

The WMH volumes were derived using probability maps, which were computed from segmented FLAIR images using the lesion prediction algorithm (LPA), implemented in the Lesion Segmentation Toolbox (LST^2^, version 2) of SPM12. As described in our previous studies (Garnier-Crussard et al., 2020, 2021; Gaubert et al., 2021), processing comprised the co-registration of individual raw FLAIR images to their respective T1-weighted image and the computation of native-space lesion probability maps. To quantify WMH volumes, native-space lesion probability maps were binarized at a threshold at 0.5 and a minimal cluster size of 10 mm^3^. Finally, global WMH volumes were derived from a brain mask of the whole cortex, while excluding ventricles, brain stem and cerebellum (Gaubert et al., 2021). This brain mask was warped from MNI to native space, using the inverse transformation matrix created during the previous normalization step within the “Segment” process of the T1-weighted images (see above). For the present analyses, the first available timepoint with a valid measurement of WMH was selected for the analyses: *n* = 71 at timepoint T1, *n* = 13 at timepoint T2, and *n* = 11 at timepoint T3. WMH volumes were adjusted for TIV to account for individual head and brain sizes.

#### Assessment of Resting-State Functional Connectivity

The RSFC analysis procedure was carried out in agreement with the previous study (Köbe et al., 2021). Preprocessing of the rs-fMRI data has been performed according to the default pipeline ‘‘direct normalization to MNI space’’ of the publicly available CONN Functional Connectivity Toolbox^3^ (version 19.c) in conjunction with SPM 12 (Whitfield-Gabrieli and Nieto-Castanon, 2012), as described previously (Benson et al., 2018). Briefly, raw functional images were motion corrected (realigned and unwarped), slice-time corrected, and co-registered to each individual T1-weighted image, before they were normalized to MNI template space and spatially smoothed (8 mm full-width-half-maximum Gaussian kernel).

Outlier scans were detected using the Artifact Detection Toolbox implemented in CONN >±3 SD in mean global intensity and frame-wise displacement exceeding 0.5 mm (combination of translational and rotational displacements). During the subsequent denoising step, physiological and movement confounds were regressed out, using a combination of component-based correction (CompCor, Behzadi et al., 2007, 5 white matter and 5 cerebral spinal fluid regressors), scrubbing (invalid outlier scans as regressors), motion regression (6 motion parameters and 6 first-order temporal derivates), and filtering (0.008 – 0.09 Hz). Thus, confounds like head motion, peripheral physiology, and other imaging artifacts were limited. Mean framewise displacement (FD), caused by in-scanner head motion, was calculated after scrubbing to be used as a covariate in the statistical analyses.

For the assessment of network-based and global (whole-brain) RSFC, we used the “Schaefer parcelation” atlas (Schaefer et al., 2018), as done in the previous study (Köbe et al., 2021). This parcelation scheme comprises 400 predefined parcels that belong to seven neocortical functional networks, namely the default mode (DMN), salience and ventral attention (SAL/VAN), fronto-parietal (FPN), limbic (LIM), dorsal attention (DAN), visual (VIS), and somatomotor network (SM). The average time series from each parcel was extracted from MNI template space, correlated with one another (Pearson) and Fisher z-transformed, using Matlab. Thus, individual correlation matrices were generated (400 × 400). Given the ambiguous interpretation of negative correlations (Murphy et al., 2009), correlation matrices were thresholded to keep only positive correlations, as done previously (Köbe et al., 2021).

Next, correlation estimates from parcels of the same network were averaged to compute the mean RSFC within each functional network. Moreover, we calculated the individual global RSFC, corresponding to the average connectivity of each parcel to all other parcels of the brain (400 × 400). We investigated both network-specific and global functional connectivity as done in the previous study (Köbe et al., 2021) given that these measures are differentially impact by the different VRF. In the current study, RSFC could be estimated for all 95 participants at T1 and T2 and for 55 of these participants (58%) additionally at T3. In Figure 1 an overview of the rs-fMRI processing and analyses is shown.

### PET Acquisition and Processing

The PET scans with florbetapir ([^18^F]AV45) were acquired on a Discovery RX VCT 64 PET-CT scanner (General Electric Healthcare, United States) at the Cyceron Center (Caen, France) with a resolution of 3.76 × 3.76 × 4.9 mm^3^ (field of view = 157 mm) to measure Aβ deposition within the brain. Approximately 4 MBq/kg of AV45 were injected intravenously and 47 planes with a voxel size of 1.95 × 1.95 × 3.2 mm^3^ were obtained at 50–70 min post-injection. A transmission scan was performed for attenuation correction before the PET acquisition.

The AV45-PET images were linearly co-registered to the corresponding T1-weighted MRI image and warped onto the MNI template, using the deformation matrices derived from the “Segment” procedure (described under MRI analyses). Standardized uptake value ratios (SUVR) were computed by dividing the cerebral tracer uptake by the tracer uptake of the whole cerebellum serving as a reference region. Mean SUVR values were extracted from brain regions typically affected in AD, using a modality-specific AD meta-signature mask, including all neocortical regions, but excluding para-hippocampi, pre- and post-central gyri, and occipital cortices (Besson et al., 2015). For the current analyses, the first available timepoint with a valid measurement of Aβ-PET SUVR was selected: *n* = 77 at T1 and *n* = 18 at T2.

### Assessment of Cognitive Performance

A comprehensive neuropsychological test battery, including tests of episodic, semantic and working memory, language skills, and executive and visuospatial functions as previously described (Besson et al., 2015), was administrated to all participants longitudinally. Participants included in the current study, underwent the cognitive assessment at least two times and up to three times across 3 years of study duration (Table 1).

**TABLE 1:** Sample characteristics at baseline.

	All *n* = 95	OA *n* = 43	SCD *n* = 18	MCI *n* = 34	Group Differences
Sex [No. women (%)]	44 (46)	23 (53)	8 (44)	13 (38)	0.405 ^I^
Age [years]	70 (6.9; 54 – 85)	70 (6.3; 60 – 84)	65 (5.7; 54 – 74)	72 (7,1; 58 – 85)	0.001 ^II a, c^
Education [years]	12.5 (3.7; 7 – 20)	12.7 (3.8; 7 – 20)	14.1 (2.7; 10 – 20)	11.2 (3.6; 7 – 20)	0.020 ^II c^
systolic blood pressure [mmHg]	144 (23; 101 – 209)	146 (20; 103 – 198)	135 (25; 101 – 189)	146 (24; 104 – 209)	0.181 ^II^
diastolic blood pressure [mmHg]	80 (13; 54 – 118)	82 (12; 60 – 118)	78 (13; 55 – 106)	80 (13; 54 – 113)	0.541 ^II^
BMI [kg/m2]	24.6 (3.7; 17 – 38)	24.3 (3.1; 19 – 34)	23.7 (3.1; 17 – 31)	25.5 (4.5; 18 – 38)	0.216 ^II^
HbA1c [%]	5.7 (0.42; 4.8 – 7.8)	5.73 (0.31; 5.2 – 6.6)	5.59 (0.27; 5.0 – 6.0)	5.76 (0.57; 4.8 – 7.8)	0.378 ^II^
WMH volume [divided by TIV]	5.39 (6.53; 0.18 – 45.7)	4.34 (4.03; 0.18 – 19.5)	4.59 (5.45; 0.54 – 16.9)	7.13 (8.98; 0.19 – 45.7)	0.403 ^II^
[^18^F]AV-45 SUVR [median (IQR; range; % of Aβ positive) individuals]	1.20 (±0.17; 0.97 – 1.89; 44)	1.20 (±0.16; 0.97 – 1.58; 35)	1.19 (±0.13; 1.12 – 1.52; 39)	1.31 (±0.46; 0.97 – 1.89; 59)	0.003 ^III b, c^
GM volume [%TIV]	44 (3.1; 33 – 50)	43.6 (2.4; 37 – 49)	45.2 (2.6; 38 – 49)	42.8 (3.8; 33 – 50)	0.032 ^II c^
Number of timepoints: rs-fMRI	2.6 (0.5; 2 – 3)	2.6 (0.5; 2 – 3)	2.7 (0.5; 2 – 3)	2.5 (0.5; 2 – 3)	0.269 ^I^
Number of timepoints: Executive Function	2.5 (0.5; 2 – 3) *n* = *88*	2.6 (0.5; 2 – 3) *n* = *42*	2.6 (0.5; 2 – 3) *n* = *18*	2.4 (0.5; 2 – 3) *n* = *28*	0.075 ^I^
Number of time points: Episodic Memory	2.6 (0.5; 2 – 3) *n* = *92*	2.6 (0.5; 2 – 3) *n* = *43*	2.7 (0.5; 2 – 3) *n* = *18*	2.4 (0.5; 2 – 3) *n* = *31*	0.162 ^I^
Number of time points: Processing Speed	2.6 (0.5; 2 – 3) *n* = *90*	2.6 (0.5; 2 – 3) *n* = *42*	2.7 (0.5; 2 – 3) *n* = *18*	2.4 (0.6; 2 – 3) *n* = *30*	0.104 ^I^
Number of time points: Working Memory	2.6 (0.5; 2 – 3) *n* = *94*	2.6 (0.5; 2 – 3) *n* = *43*	2.7 (0.5; 2 – 3) *n* = *18*	2.5 (0.5; 2 – 3) *n* = *33*	0.226 ^I^

*Numbers are given if applicable as mean, standard deviation, and range (parenthesis). The sample size is provided if different from sample size specified in first row. I = Chi-Square^/^II = ANOVA/III = Kruskal-Wallis test: significant group differences between ^a^OA vs. SCD, ^b^OA vs. MCI, ^c^SCD vs. MCI. BMI, HbA1c and WMH volumes were log-transformed before statistical group comparisons. The WMH volume was assessed at time point T1 for n = 71, at T2 for n = 13 and at T3 for n = 11 participants. The Aβ-PET SUVR was assessed at time point T1 for n = 77 and at T2 for n = 18 participants and a global Aβ positivity threshold of 1.22 was used to define Aβ+ individuals. BMI, body-mass-index; HbA1c, glycated hemoglobin A1; WMH, white matter hyperintensities; AV-45 SUVR, florbetapir standard uptake value ratio; GM, gray matter; TIV, total intracranial volume.*

To obtain robust proxies of domain-sensitive cognitive performance, four individual cognitive composite scores were calculated, assessing executive function, episodic memory, processing speed, and working memory, as described in our previous study (Garnier-Crussard et al., 2020). To create composite scores, the individual cognitive scores were z-transformed (i.e., using OA of the IMAP+ sample as the reference group) and averaged. Composite scores were computed in participants with at least two corresponding individual scores. Z-scores derived from reaction times (i.e., TMT and Stroop) were reversed before being averaged, such that higher values always indicated better performance.

In brief, the executive function composite included the phonemic verbal fluency test score (Letter P), a flexibility score calculated on the Trail Making Test (TMT) (time difference between parts B and A divided by time of part A), and a score of inhibition computed from the Stroop test (time difference between interference and naming tasks) (Godefroy, 2008). The episodic memory composite included immediate free recalls and delayed free recall of the Free and Cued Selective Reminding Test (FCSRT) (Grober et al., 1988), memory subscore of the Mattis Dementia Rating Scale (Mattis, 1976), and free recalls of the “encoding, storage, retrieval” paradigm (Eustache et al., 2015). The processing speed composite included the TMT part A score (times), and color reading and color naming of the Stroop test (Godefroy, 2008). The working memory composite included scores of forward and backward digit span (Petermann and Wechsler, 2012).

### Statistical Analyses

Statistical analyses were performed with R 4.0.3 and R-Studio 1.3.1093 (The R Foundation). Two-sided *P* values less than 0.05 were considered to be statistically significant. The statistical analysis procedure was carried out in close correspondence with the previous study (Köbe et al., 2021). Overall, effect sizes were expected to be subtle and the previous findings (Köbe et al., 2021) clearly supported *a priori* hypotheses with respect to higher-order brain networks that are susceptible to VRF, aging and AD, thus no corrections for multiple comparisons were applied.

Our total sample was first characterized by demographic factors, VRF levels and biomarkers of brain pathology. Baseline differences between diagnostic groups (OA, SCD, and MCI) were investigated using χ^2^ (Chi-Square) tests for categorical variables. One-way analyses of variance (ANOVA) or Kruskal-Wallis test were used for comparisons of parametric and non-parametric continuous variables, respectively, followed by Tukey’s multiple comparisons tests and Wilcoxon rank sum tests for corresponding pairwise comparisons. Visual inspection for approximate symmetric distribution of the data was done on QQ-plots. For descriptive purpose, abnormal levels of VRF were indicated for all participants using established cut-off thresholds (World Health Organization, 2000, 2006; Whelton et al., 2018). For all statistical analyses, the measures of BMI, HbA1c and WMH volumes were log-transformed to reduce skewness and to achieve a near normal distribution. Subsequent analyses were conducted across all diagnostic groups to map the anticipated trajectories along the cognitive continuum with range of older people free of dementia, but some of whom are at increased risk.

We ran linear mixed-effects models (LME, lmer function of the lme4 package) (Bates et al., 2015) to characterize the overall change in network/global RSFC over up to three years, and its relation to peripheral VRF (i.e., systolic and diastolic blood pressure, BMI, HbA1c), WMH volume and brain Aβ deposition. All of these independent variables of interest showed no multi-collinearity (variance inflation factor < 5) and were therefore entered in the same statistical model. Next to these variables of interest, baseline age, sex, gray matter volume (adjusted for TIV), diagnostic status and mean FD at each visit were included as potential confounding factors (covariates). Follow-up time of rs-fMRI was operationalized individually as years from baseline measurement (below referred to as: time). The following equation was used for these models: longitudinal network/global RSFC ∼ systolic blood pressure × time + diastolic blood pressure × time + BMI × time + HbA1c × time + WMH × time + Aβ-PET SUVR × time + covariates × time. Selected interaction effects were graphed to evaluate the directionality of the associations. Continuous measures were thereby divided into tertiles, only for the purpose of improved visualization.

We also investigated, if significant associations between the VRF of interest and changes in RSFC over time were moderated by Aβ burden. Therefore, we dichotomized participants with an Aβ-PET SUVR ≥ 1.22 into Aβ-positive individuals and < 1.22 into Aβ-negative individuals, using thresholds that were established and described in our previous study (Vanhoutte et al., 2021). Then the models described above were rerun, except that the term “Aβ-PET SUVR × time” was replaced by a 3-way interaction between the vascular risk factor of interest (i.e., that was significantly associated with RSFC in the first models), Aβ positivity status and time.

Finally, an exploratory analysis was conducted to assess whether or not changes in RSFC that were significantly related to our VRF measures, were linked to changes in individual cognitive performance over time. We thus extracted the predicted slopes of RSFC (change over time) from the first LME models (see above) and included these as dependent variables, as well as their interaction with time, in subsequent LME models. Longitudinal cognitive performance was operationalized by four composite scores (described above). Age, sex, education and their interaction with time were included as covariates. The following base equation was applied: longitudinal cognition ∼ RSFC slope × time + covariates × time. Follow-up time of cognition was operationalized individually as years from baseline cognitive assessment.

In all LME models, we included individual RSFC or cognition intercepts and slopes as random effects long with the fixed effects of variables of interest (i.e., VRF, WMH, Aβ-PET SUVR, and RSFC slopes) and covariates. Models included both all main effects as well as their interactions with time. All continuous variables were z-transformed prior to model estimation. All models used the restricted maximum likelihood method and were fit with an unstructured variance-covariance and type III sum of squares. Denominator degrees of freedom were calculated with the Satterthwaite approximations.

## Results

### Sample Characteristics

Participant characteristics are summarized in Table 1. Diagnostic groups were comparable regarding sex distribution, blood pressure (systolic and diastolic), BMI, HbA1c, WMH volumes and assessment timepoints of rs-fMRI (all *p’s* ≥ 0.1). There were significant differences in age and education across diagnostic groups (ANOVA: *p’s* < 0.05). The MCI patients showed a higher mean Aβ-PET SUVR (Kruskal-Wallis: *p* = 0.003; OA vs. MCI *p* = 0.003; SCD vs. MCI *p* = 0.036) and lower GM volume (ANOVA: *p* = 0.032; SCD vs. MCI *p* = 0.024). Across the total sample, abnormal levels of VRF were recorded for systolic blood pressure (≥130 mmHg, *n* = 70 [74%]), diastolic blood pressure (≥80 mmHg, *n* = 45 [47%]), body mass index (≥30 kg/m^2^, *n* = 8 [6.5%]) and HbA1c (≥ 6.5%, *n* = 3 [3%]).

### Longitudinal Change in Resting-State Functional Connectivity and Its Association With Vascular Risk Factors, White Matter Hyperintensity, and Abeta Load

Across the entire sample and time, we found an overall increase in RSFC over time within the DMN (β = 0.060, standard error [SE] = 0.026, *t* = 2.31, *p* = 0.024), the SAL/VAN (β = 0.069, SE = 0.024, *t* = 2.86, *p* = 0.005), FPN (β = 0.095, SE = 0.030, *t* = 3.13, *p* = 0.002), VIS (β = 0.090, SE = 0.037, *t* = 2.42, *p* = 0.018) networks and in global RSFC (β = 0.047, SE = 0.023, *t* = 2.05, *p* = 0.043), adjusted for age, sex, diagnosis and mean FD (Table 2, extended table is provided in the Supplementary Material in Supplementary Table S1). No significant overall increase in RSFC over time was observed within the LIM, DAN and SM networks (all *p’s* ≥ 0.05).

**TABLE 2:** Longitudinal change in RSFC within networks and its associations with VRF, WMH, and Aβ.

	DMN				SAL/VAN				FPN				LIM				DAN				VIS				SM				Global			
No. of obs. = 865																																
No. of part. = 247	Est.	SE	t	p	Est.	SE	t	p	Est.	SE	t	p	Est.	SE	t	p	Est.	SE	t	p	Est.	SE	t	p	Est.	SE	t	p	Est.	SE	t	p
Time	0.0595	0.026	2.308	0.024*	0.0691	0.024	2.858	0.005**	0.0955	0.030	3.131	0.002**	0.0094	0.026	0.366	0.716	0.0356	0.032	1.099	0.275	0.0896	0.037	2.418	0.018*	0.0324	0.023	1.414	0.161	0.0467	0.023	2.050	0.043*
sBP × time	0.0269	0.017	1.594	0.116	0.0238	0.016	1.507	0.136	0.0320	0.020	1.595	0.115	0.0269	0.017	1.601	0.115	0.0182	0.021	0.851	0.397	0.0172	0.024	0.710	0.480	0.0024	0.015	0.161	0.873	0.0178	0.015	1.186	0.239
dBP × time	–0.0335	0.016	–2.051	0.044*	–0.0402	0.015	–2.629	0.010*	–0.0414	0.019	–2.138	0.036*	–0.0215	0.016	–1.319	0.192	–0.0227	0.021	–1.100	0.275	–0.0347	0.024	–1.477	0.144	–0.0074	0.015	–0.505	0.615	–0.0255	0.015	–1.760	0.082.
BMI × time	–0.0016	0.014	–0.113	0.910	0.0077	0.013	0.581	0.563	0.0140	0.017	0.836	0.406	0.0060	0.014	0.420	0.676	–0.0200	0.018	–1.119	0.266	–0.0162	0.021	–0.787	0.434	–0.0174	0.013	–1.385	0.170	–0.0096	0.012	–0.770	0.443
HbA1c × time	–0.0170	0.015	–1.148	0.254	–0.0294	0.014	–2.117	0.037*	–0.0232	0.017	–1.327	0.188	–0.0201	0.015	–1.344	0.183	–0.0188	0.019	–1.001	0.319	–0.0394	0.022	–1.812	0.073.	–0.0077	0.013	–0.585	0.560	–0.0181	0.013	–1.391	0.167
WMH × time	0.0272	0.018	1.518	0.133	0.0174	0.017	1.038	0.303	0.0312	0.021	1.471	0.145	0.0081	0.018	0.451	0.654	0.0070	0.023	0.310	0.758	0.0232	0.026	0.900	0.371	0.0048	0.016	0.301	0.764	0.0124	0.016	0.781	0.437
Aβ × time	–0.0127	0.016	–0.813	0.418	–0.0152	0.015	–1.035	0.304	–0.0236	0.018	–1.280	0.204	–0.0113	0.016	–0.722	0.473	–0.0019	0.020	–0.095	0.925	–0.0232	0.023	–1.021	0.311	0.0085	0.014	0.613	0.541	–0.0056	0.014	–0.403	0.688

Using linear mixed-effects models, we assessed resting-state functional connectivity (RSFC) trajectories across the entire study sample within seven functional networks as defined by the Schaefer parcelation atlas (DMN, default mode network; SAL/VAN, salience and ventral attention network; FPN, fronto-parietal network; LIM, limbic network; DAN, dorsal attention network; VIS, visual network and SM, somatomotor network) and throughout the whole brain (global). We investigated the associations between vascular risk factors (VRF; systolic blood pressure, sBP; diastolic blood pressure, dBP; body-mass-index, BMI; glycated hemoglobin A1, HbA1c), white matter hyperintensities (WMH), and Amyloid-β (Aβ) and longitudinal changes in RSFC over time. A random effects intercept and slope of RSFC for each individual were included in the models. Models were corrected for baseline age, sex, gray matter volume (GMV), diagnostic group, for longitudinal mean frame-displacement (FD) and for the interactions of selected covariates with time. Unstandardized estimates (Est.), standard errors (SE), T values and P values (**p < 0.01, *p < 0.05) as well as marginal (R2m) and conditional (R2c) R^2^ values are presented. Other abbreviations: Obs., observations; part, participants.

**FIGURE 2:**
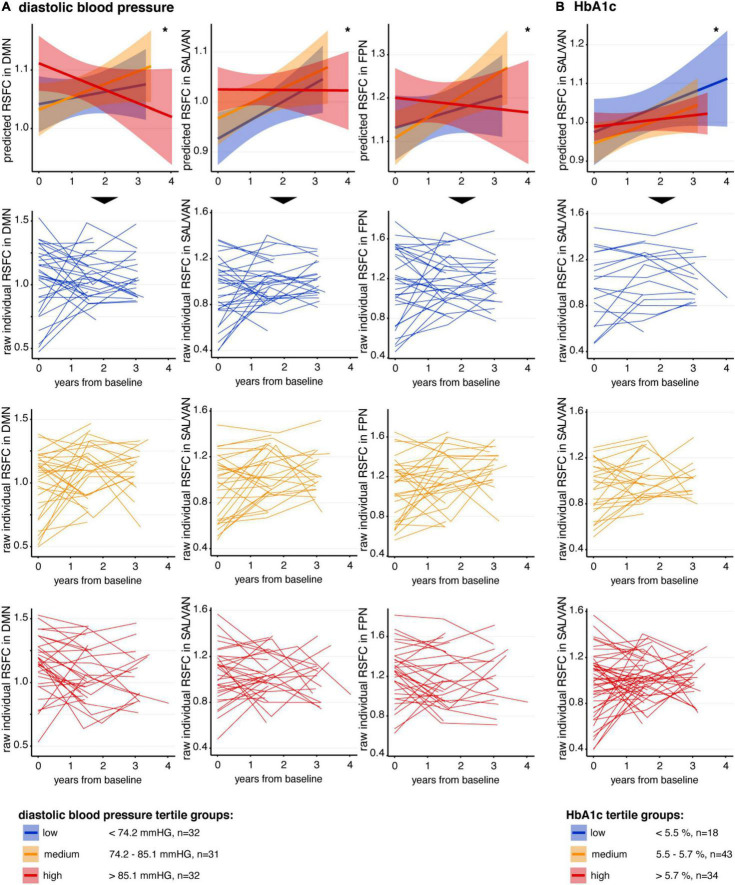
Association between VRF and longitudinal change in RSFC. In the first row of panels, mean predicted RSFC estimates from baseline to follow-ups, derived from linear-mixed effects models, are plotted for descriptive visualization using tertiles of low (blue), medium (orange), and high (red) **(A)** diastolic blood pressure and **(B)** HbA1c, respectively. The small lines in the lower three rows of panels represent individual raw RSFC trajectories. Continuous measures of diastolic blood pressure and HbA1c were divided into tertiles only for better descriptive visualization of significant interactions. Shaded regions represent 95% confidence intervals. DMN, default mode network; RSFC, resting-state functional connectivity; SAL/VAN, salience and ventral attention network. *p < 0.05.

Next, we assessed how longitudinal change in RSFC was related to VRF. We found negative interactions for diastolic blood pressure (dBP × time) and for that HbA1c (HbA1c × time). Visualization of these significant interactions (Figure 2) indicated that higher levels of diastolic blood pressure were more likely to be associated with a decrease of RSFC over time (i.e., a reduction of slope) within the DMN (β = –0.034, SE = 0.016, *t* = –2.05, *p* = 0.044), the SAL/VAN (β = –0.040, SE = 0.015, *t* = –2.63, *p* = 0.010), and the FPN (β = –0.041, SE = 0.019, *t* = –2.14, *p* = 0.036) (Figure 2 and Table 2). Higher HbA1c levels were rather associated with a reduction of the observed RSFC increase over time within the SAL/VAN (β = –0.029, SE = 0.014, *t* = –2.12, *p* = 0.037; Figure 2 and Table 2). Longitudinal changes in RSFC were not significantly associated with systolic blood pressure, BMI, or WMH volume in our sample (all *p*’s ≥ 0.05, Table 2).

Regarding Aβ pathology, there was no significant association between global Aβ deposition and change in RSFC over time in the specific networks and in global RSFC (all *p’s* ≥ 0.05, Table 2). Also, the Aβ positivity status did not significantly moderate the observed significant associations between diastolic blood pressure and RSFC changes over time (interaction: Aβ positivity × diastolic blood pressure × time on longitudinal RSFC in the DMN, SAL/VAN, and FPN: all *t*’s ≤ 1.606 and *p’s* ≥ 0.112) or HbA1c and RSFC changes over time (interaction: Aβ positivity × HbA1c × time on longitudinal RSFC in SAL/VAN: *t* = –1.129 and *p* = 0.262) (data not shown).

### Association Between Changes in Resting-State Functional Connectivity and Changes in Cognitive Performance

We further explored whether or not the VRF-related changes in RSFC trajectories (modeled by slopes of significant predicted LMEs) were associated with longitudinal changes in cognitive performance. No significant associations were found between the RSFC slopes of the DMN, SAL/VAN and FPN and cognitive changes in the four cognitive composites over time, adjusted for age, sex and education (*p’s* ≥ 0.2, Supplementary Table S2).

## Discussion

### Summary of Results

The main goal of this study was to replicate and expand earlier findings on the impact of VRF on RSFC trajectories within intrinsic brain networks (Köbe et al., 2021). Maintaining the data analysis strategy, the current results were derived from the independent IMAP+ cohort (e.g., Mutlu et al., 2017; Gaubert et al., 2021). (1) Our results show that VRF were independently associated with RSFC changes over time within higher-order brain networks. This finding was replicated for diastolic blood pressure. (2) The VRF-related associations were independent of pathological brain burden and no comparable effects of brain pathology were observed. This finding was replicated for Aβ load. (3) Our study expands earlier findings by three important novel aspects: We document that higher HbA1c levels were independently associated with RSFC changes over time. We clarify that the VRF-related associations were independent of WMH volumes, which had no measurable effect on RSFC changes. Finally, there were no detectable associations between the VRF-related changes in RSFC and longitudinal changes in cognitive performance. Altogether, our findings substantiate a presumed causal role of VRF in RSFC changes over time within cognitive networks, namely the DMN, SAL/VAN and FPN, known to be affected by aging and AD.

### Replication of Findings

The present study largely confirms that VRF can be independently associated with RSFC changes over time, involving higher-order brain networks pertinent to cognitive health. First, we replicated earlier findings showing that there is an overall increase in RSFC over time in our sample of non-demented older adults – both within global RSFC and within the DMN, SAL/VAN and FPN networks. The effect has been interpreted to reflect some form of hyper-connectivity that mirrors earlier observations by Köbe et al. (2021) and other longitudinal findings, proposing that an initial increase in RSFC occurs in older adults until about ∼74 years of age, which is followed by a decline of RSFC as age progresses (Ng et al., 2016; Staffaroni et al., 2018). Similar to the previous study (Köbe et al., 2021), our current sample of older adults is relatively young, thus potentially explaining the observed increase of RSFC over time. Moreover, it appears that the cascading network failure may be hastened in the presence of VRF, as specifically indicated by the negative interactions between specific VRF and changes in RSCF over time. Namely, higher diastolic blood pressure levels and higher HbA1c levels were independently associated with RSFC changes over time in the DMN, the FPN, and/or the SAL/VAN, with the latter brain network showing an overlapping effect of the two VRF. Interestingly, out of the seven intrinsic brain networks, those displaying a functional vulnerability to VRF are also targeted by aging and AD (Buckner et al., 2008; Betzel et al., 2014; Elman et al., 2016; Grady et al., 2016; Badhwar et al., 2017). In contrast, we did not find such effects in the non-cognitive visual (VIS) and somatomotor (SM) networks.

As an important outcome, we thus reproduced the previous findings (Köbe et al., 2021) in the PREVENT-AD cohort (Breitner et al., 2016) using a highly similar methodological approach in the independent IMAP+ cohort. Both cohorts are comparable in the sense that they provide monocentric, longitudinal, and multimodal (i.e., clinical, behavioral and neuroimaging) data in older participants at increased risk for developing AD. Previously, it has been shown that higher levels of blood cholesterol and diastolic blood pressure were associated with a reduction of RSFC over time in the DMN and in global RSFC, respectively (Köbe et al., 2021). Together, the earlier and present longitudinal findings converge very well with the existing (mostly cross-sectional) studies, indicating a greater vulnerability of higher-order brain networks to a variety of VRF including higher levels of blood pressure, cholesterol, and HbA1c as well as the metabolic syndrome (Li et al., 2015; Zhang et al., 2016; Rashid et al., 2019; Feng et al., 2020; Veldsman et al., 2020). As in the previous study (Köbe et al., 2021), a similar association was not found for BMI. The BMI is proposed to influence VRF related to diabetes and hypertension (Gill et al., 2021), which may be more closely associated with RSFC alterations in older adults (see below).

### Novel Findings

Our current results add important novel aspects hinting at a causal role of VRF, namely diastolic blood pressure and HbA1c, in RSFC changes over time. More specifically, we document that higher diastolic blood pressure levels were more likely to be associated with a decrease of RSFC over time in the DMN, FPN, and SAL/VAN. In addition, higher HbA1c levels were rather associated with a reduction of the observed RSFC increase in the SAL/VAN, thus pinpointing the latter network as a common target point. These findings mirror cross-sectional findings, showing that hypertension and higher HbA1c levels are associated with altered RSFC in distributed brain networks at the population level (Feng et al., 2020) and in patients with diabetes (Zhang et al., 2020), respectively. The broad pattern of RSFC disturbances may reflect an overall disruption of microvascular structure as previously linked to diastolic hypertension (Caunca et al., 2020) and long-term glycemic exposure (Samanta, 2021).

Importantly, it is shown here that the impact of VRF on RSFC changes over time was largely independent of concomitant pathology, as measured using Aβ load and WMH. While this was formerly shown for Aβ deposition (Köbe et al., 2021), here we extend these findings to WMH, as a manifestation of cerebral small vessel disease (Wardlaw et al., 2013; Prins and Scheltens, 2015). It is likely that VRF work through various mechanisms that adversely impact brain and cognitive health (Villeneuve and Jagust, 2015; Vemuri et al., 2022) and are not fully captured by the present neuroimaging biomarkers of brain pathology. The observed effects of VRF may involve other microvascular lesions such as lacunes, microbleeds and microinfarcts (Wardlaw et al., 2013; Van Veluw et al., 2017) or other cerebrovascular mechanisms such as changes in cerebral blood flow or blood-brain barrier permeability (Zlokovic, 2011; de la Torre, 2012). The latter mechanisms are proposed to promote neural damage that is not dependent on Aβ pathology and vascular brain lesions (Villeneuve and Jagust, 2015), warranting further investigation.

### Implications of Our Findings

The fact that poor vascular health may hasten RSFC changes within cognitive networks is important, because such functional brain alterations are implicated in AD pathogenesis – even at preclinical stages. While this was mainly shown for the DMN (Buckner et al., 2009), recent observations in normal older adults clearly demonstrate that lower RSFC in the DMN, SAL and FPN networks are predictive of cognitive decline over time in synergy with Aβ deposition (Buckley et al., 2017). By promoting early RSFC alterations within the very same brain networks, VRF may perhaps deplete cognitive reserve capacities (Franzmeier et al., 2017; Benson et al., 2018) and thereby accelerate cognitive disturbances in the setting of Aβ. In a recent framework functional connectivity is proposed as a candidate substrate for cognitive reserve, meaning that adaptive functional brain processes may help counteract brain pathology and damage in older age (Stern et al., 2020). In this line, RSFC alterations within higher-order brain networks, as associated with VRF, could perhaps contribute to limited cognitive reserve in late life and increase the vulnerability to aging and AD. Higher levels of diastolic blood pressure and HbA1c have also been linked to Aβ pathology, AD-typical neurodegeneration, and/or cerebrovascular disease (Byun et al., 2017; McNeil et al., 2017; Caunca et al., 2020; Garfield et al., 2021). Together, the findings converge on the view that good vascular health may help preserve brain health and cognitive resilience in older adults (de la Torre, 2010).

### Negative (Null) Results

We did not detect any significant associations between pathological brain burden and changes in RSFC trajectories within the intrinsic brain networks. This observation replicates the previous study (Köbe et al., 2021) and is in part convergent with other reports (Tahmi et al., 2020). Cross-sectional studies have, however, documented RSFC alterations in the setting of Aβ burden or vascular brain injury in non-clinical and clinical cohorts (Hedden et al., 2009; Mormino et al., 2009; Elman et al., 2016; Schulz et al., 2021), with some of the Aβ-related RSFC alterations involving more focal hub regions (Hedden et al., 2009; Mormino et al., 2011). In addition, we did not detect a cognitive relevance of the VRF-related changes in RSFC alterations over time, albeit this was shown in a recent cross-sectional study on hypertension (Feng et al., 2020). It might be possible that pathological levels of VRF are more closely associated with RSFC alterations of cognitive relevance. Alternatively, VRF may foster vascular brain injury (van Dijk et al., 2008), which is reliably associated with lower cognitive functioning in older adults of the IMAP+ cohort (Garnier-Crussard et al., 2020) and other studies (Kloppenborg et al., 2014; Vemuri et al., 2015; Benson et al., 2018). Given our current null results, the cognitive relevance of the VRF-related changes in RSFC trajectories over time remains to be established.

### Strength and Limitation

The present study has strengths and limitations. Our current findings provide supporting evidence for a critical role of vascular health on RSFC within higher-order brain networks in older adults. One particular strength of our study is the longitudinal study design, allowing to investigate, replicate and extend earlier findings on the impact of VRF on changes in RSFC over time (Köbe et al., 2021) using an independent monocentric cohort of well-described older participants. Thanks to this unique and high-quality data pool, we additionally adjusted for measures of brain pathological burden and incorporated longitudinal cognitive assessments in our analyses. We feel that the present combination of well-characterized data outweighs some of the limitations of our study design including the limited sample size. Knowledge about the role of vascular health in brain aging is a key factor in the development of preventative strategies.

Several limitations need to be considered, when interpreting our results. (1) The majority of VRF were assessed at baseline with only one measurement available, potentially limiting the reliability of our markers. (2) We had a limited amount of VRF available. Therefore, potential effects of e.g., cholesterol levels on RSFC changes over time seen in the previous study (Köbe et al., 2021) could not be replicated. (3) It would be important to consider the effects of cardiovascular medication in the observed associations. There was no detailed information on medication intake in the present cohort. However, similar results as shown by the present study were found with respective adjustments for vascular medication in Köbe et al. (2021). (4) We merged participants along the cognitive continuum with normal cognition, SCD, and MCI in the attempt to enhance statistical power and variance. On the downside, this approach may have obscured group-specific associations, necessitating further investigation with larger sample sizes. (5) The current sample of older adults was mildly affected by vascular risk, especially for the BMI and HbA1c measures, comparable to the previous study (Köbe et al., 2021). It is nonetheless important to document the impact of VRF on RSFC in higher-order brain networks, even if subtle. (6) Having replicated previous findings assessing the association between VRF and within-network RSFC changes over time, there is a need to assess the link between VRF and changes in between-network RSFC in prospective studies. Overall, it could be assumed that functional brain alterations associated with VRF may become more relevant to cognitive health as pathological burden accumulates.

## Conclusion

Our present findings in the independent IMAP+ cohort indicate that VRF related to higher diastolic blood pressure levels and higher HbA1c levels promote alterations in RSFC over time within higher-order brain networks. This observed impact of vascular health could potentially limit cognitive reserve capacities in older adults and thus increase the vulnerability to aging and AD. Further work is needed to establish the cognitive relevance of the VRF-related changes in large-scale functional connectivity patterns.

## Data Availability Statement

The data analyzed in this study is subject to the following licenses/restrictions: The dataset analyzed in the present study is not publicly available due to data protection regulations. It is available from the corresponding author (GC) on reasonable request. Existing data analysis packages were used for statistical analyses. Respective scripts are available from the corresponding author (MW) on reasonable request. Requests to access these datasets should be directed to MW, miranka.wirth@dzne.de; GC, chetelat@cyceron.fr.

## Ethics Statement

The study involved human participants and was reviewed and approved by the Comité de Protection des Personnes Nord-Ouest III. The patients/participants provided their written informed consent to participate in this study.

## Author Contributions

TK, GC, and MW contributed to study concept and design. JG, RF, and BL took part in data acquisition and processing as well as quality check control. MG, TK, AG-C, CL, GC, and MW carried out data analysis and interpretation and drafted and revised the manuscript. JG, RF, and VS took part in participants’ recruitment and selection process, clinical evaluation, and monitoring of participants. All authors read and approved the final version.

## Conflict of Interest

The authors declare that the research was conducted in the absence of any commercial or financial relationships that could be construed as a potential conflict of interest.

## Publisher’s Note

All claims expressed in this article are solely those of the authors and do not necessarily represent those of their affiliated organizations, or those of the publisher, the editors and the reviewers. Any product that may be evaluated in this article, or claim that may be made by its manufacturer, is not guaranteed or endorsed by the publisher.

## Footnotes

1
www.fil.ion.ucl.ac.uk/spm

2
www.statistical-modelling.de/lst.html

3
www.nitrc.org/projects/conn
