# Supplementary material for: Vascular Health Is Associated With Functional Connectivity Decline in Higher-Order Networks of Older Adults
Source: Front Integr Neurosci. 2022 Apr 26;16:847824. doi: 10.3389/fnint.2022.847824 (PMC9088922; doi:10.3389/fnint.2022.847824)
Supplement: Supplementary file 1 [file Table_1.docx]

**Vascular health is associated with functional connectivity decline in higher-order networks of older adults**

Miranka Wirth, PhD; Malo Gaubert, MSc; Theresa Köbe, PhD; Antoine Garnier-Crussard, MD, MSc; Catharina Lange, PhD; Julie Gonneaud, PhD; Robin de Flores, PhD; Brigitte Landeau, MSc; Vincent de la Sayette, MD; Gaël Chételat, PhD

## Table S1. Longitudinal change in RSFC within networks and its associations with VRF, WMH, and Aβ

|  | **DMN** |  |  |  |  | **SAL/**  **VAN** |  |  |  |  | **FPN** |  |  |  |  | **LIM** |  |  |  |  | **DAN** |  |  |  |  | **VIS** |  |  |  |  | **SM** |  |  |  |  | **global** |  |  |  |  |
| --- | --- | --- | --- | --- | --- | --- | --- | --- | --- | --- | --- | --- | --- | --- | --- | --- | --- | --- | --- | --- | --- | --- | --- | --- | --- | --- | --- | --- | --- | --- | --- | --- | --- | --- | --- | --- | --- | --- | --- | --- |
| No. of obs. = 865 |  |  |  |  |  |  |  |  |  |  |  |  |  |  |  |  |  |  |  |  |  |  |  |  |  |  |  |  |  |  |  |  |  |  |  |  |  |  |  |  |
| No. of part. = 247 | **Est.** | **SE** | **t** | **p** |  | **Est.** | **SE** | **t** | **p** |  | **Est.** | **SE** | **t** | **p** |  | **Est.** | **SE** | **t** | **p** |  | **Est.** | **SE** | **t** | **p** |  | **Est.** | **SE** | **t** | **p** |  | **Est.** | **SE** | **t** | **p** |  | **Est.** | **SE** | **t** | **p** |  |
| Time | 0.0595 | 0.026 | 2.308 | 0.024 | * | 0.0691 | 0.024 | 2.858 | 0.005 | ** | 0.0955 | 0.030 | 3.131 | 0.002 | ** | 0.0094 | 0.026 | 0.366 | 0.716 |  | 0.0356 | 0.032 | 1.099 | 0.275 |  | 0.0896 | 0.037 | 2.418 | 0.018 | * | 0.0324 | 0.023 | 1.414 | 0.161 |  | 0.0467 | 0.023 | 2.050 | 0.043 | * |
| sBP × time | 0.0269 | 0.017 | 1.594 | 0.116 |  | 0.0238 | 0.016 | 1.507 | 0.136 |  | 0.0320 | 0.020 | 1.595 | 0.115 |  | 0.0269 | 0.017 | 1.601 | 0.115 |  | 0.0182 | 0.021 | 0.851 | 0.397 |  | 0.0172 | 0.024 | 0.710 | 0.480 |  | 0.0024 | 0.015 | 0.161 | 0.873 |  | 0.0178 | 0.015 | 1.186 | 0.239 |  |
| dBP × time | -0.0335 | 0.016 | -2.051 | 0.044 | * | -0.0402 | 0.015 | -2.629 | 0.010 | * | -0.0414 | 0.019 | -2.138 | 0.036 | * | -0.0215 | 0.016 | -1.319 | 0.192 |  | -0.0227 | 0.021 | -1.100 | 0.275 |  | -0.0347 | 0.024 | -1.477 | 0.144 |  | -0.0074 | 0.015 | -0.505 | 0.615 |  | -0.0255 | 0.015 | -1.760 | 0.082 | . |
| BMI × time | -0.0016 | 0.014 | -0.113 | 0.910 |  | 0.0077 | 0.013 | 0.581 | 0.563 |  | 0.0140 | 0.017 | 0.836 | 0.406 |  | 0.0060 | 0.014 | 0.420 | 0.676 |  | -0.0200 | 0.018 | -1.119 | 0.266 |  | -0.0162 | 0.021 | -0.787 | 0.434 |  | -0.0174 | 0.013 | -1.385 | 0.170 |  | -0.0096 | 0.012 | -0.770 | 0.443 |  |
| HbA1c × time | -0.0170 | 0.015 | -1.148 | 0.254 |  | -0.0294 | 0.014 | -2.117 | 0.037 | * | -0.0232 | 0.017 | -1.327 | 0.188 |  | -0.0201 | 0.015 | -1.344 | 0.183 |  | -0.0188 | 0.019 | -1.001 | 0.319 |  | -0.0394 | 0.022 | -1.812 | 0.073 | . | -0.0077 | 0.013 | -0.585 | 0.560 |  | -0.0181 | 0.013 | -1.391 | 0.167 |  |
| WMH × time | 0.0272 | 0.018 | 1.518 | 0.133 |  | 0.0174 | 0.017 | 1.038 | 0.303 |  | 0.0312 | 0.021 | 1.471 | 0.145 |  | 0.0081 | 0.018 | 0.451 | 0.654 |  | 0.0070 | 0.023 | 0.310 | 0.758 |  | 0.0232 | 0.026 | 0.900 | 0.371 |  | 0.0048 | 0.016 | 0.301 | 0.764 |  | 0.0124 | 0.016 | 0.781 | 0.437 |  |
| Aβ × time | -0.0127 | 0.016 | -0.813 | 0.418 |  | -0.0152 | 0.015 | -1.035 | 0.304 |  | -0.0236 | 0.018 | -1.280 | 0.204 |  | -0.0113 | 0.016 | -0.722 | 0.473 |  | -0.0019 | 0.020 | -0.095 | 0.925 |  | -0.0232 | 0.023 | -1.021 | 0.311 |  | 0.0085 | 0.014 | 0.613 | 0.541 |  | -0.0056 | 0.014 | -0.403 | 0.688 |  |
| age × time | -0.0202 | 0.021 | -0.961 | 0.340 |  | 0.0120 | 0.020 | 0.607 | 0.545 |  | -0.0162 | 0.025 | -0.648 | 0.519 |  | 0.0114 | 0.021 | 0.544 | 0.589 |  | -0.0247 | 0.026 | -0.934 | 0.353 |  | -0.0242 | 0.030 | -0.806 | 0.423 |  | 0.0057 | 0.019 | 0.303 | 0.762 |  | -0.0070 | 0.019 | -0.377 | 0.708 |  |
| sex (women to men) × time | -0.0298 | 0.026 | -1.141 | 0.257 |  | -0.0074 | 0.024 | -0.304 | 0.762 |  | -0.0535 | 0.031 | -1.729 | 0.088 | . | 0.0318 | 0.026 | 1.216 | 0.228 |  | -0.0159 | 0.033 | -0.484 | 0.630 |  | -0.0798 | 0.038 | -2.123 | 0.037 | * | -0.0258 | 0.023 | -1.106 | 0.272 |  | -0.0270 | 0.023 | -1.168 | 0.246 |  |
| GM volume × time | 0.0425 | 0.020 | 2.171 | 0.032 | * | 0.0339 | 0.019 | 1.825 | 0.071 | . | 0.0499 | 0.023 | 2.146 | 0.034 | * | 0.0208 | 0.020 | 1.055 | 0.294 |  | 0.0191 | 0.024 | 0.794 | 0.429 |  | 0.0636 | 0.028 | 2.310 | 0.023 | * | 0.0342 | 0.017 | 1.990 | 0.049 | * | 0.0369 | 0.017 | 2.152 | 0.034 | * |
| mean FD × time | 0.0089 | 0.013 | 0.665 | 0.508 |  | 0.0104 | 0.013 | 0.814 | 0.418 |  | 0.0051 | 0.016 | 0.315 | 0.753 |  | 0.0019 | 0.014 | 0.137 | 0.892 |  | 0.0242 | 0.016 | 1.485 | 0.140 |  | 0.0158 | 0.019 | 0.852 | 0.396 |  | 0.0130 | 0.012 | 1.112 | 0.269 |  | 0.0146 | 0.012 | 1.251 | 0.213 |  |
| diagnostic (SCD to OA) × time | -0.0969 | 0.035 | -2.777 | 0.007 | ** | -0.0907 | 0.033 | -2.773 | 0.007 | ** | -0.1125 | 0.041 | -2.722 | 0.008 | ** | -0.0217 | 0.035 | -0.623 | 0.535 |  | -0.1089 | 0.044 | -2.466 | 0.016 | * | -0.1756 | 0.050 | -3.493 | 0.001 | *** | -0.0550 | 0.031 | -1.759 | 0.083 | . | -0.0885 | 0.031 | -2.852 | 0.006 | ** |
| diagnostic (MCI to OA) × time | -0.0264 | 0.030 | -0.869 | 0.388 |  | -0.0468 | 0.029 | -1.636 | 0.106 |  | -0.0306 | 0.036 | -0.845 | 0.401 |  | -0.0145 | 0.030 | -0.478 | 0.635 |  | -0.0307 | 0.038 | -0.802 | 0.425 |  | -0.0573 | 0.043 | -1.319 | 0.191 |  | -0.0199 | 0.027 | -0.731 | 0.467 |  | -0.0279 | 0.027 | -1.033 | 0.305 |  |
| sBP | -0.0416 | 0.023 | -1.834 | 0.070 | . | -0.0339 | 0.024 | -1.383 | 0.170 |  | -0.0574 | 0.031 | -1.874 | 0.065 | . | -0.0207 | 0.026 | -0.803 | 0.425 |  | -0.0127 | 0.021 | -0.597 | 0.552 |  | 0.0126 | 0.025 | 0.515 | 0.608 |  | 0.0016 | 0.017 | 0.094 | 0.926 |  | -0.0161 | 0.018 | -0.914 | 0.363 |  |
| dBP | 0.0306 | 0.022 | 1.421 | 0.159 |  | 0.0425 | 0.023 | 1.830 | 0.071 | . | 0.0415 | 0.029 | 1.430 | 0.156 |  | 0.0345 | 0.024 | 1.408 | 0.163 |  | -0.0059 | 0.020 | -0.290 | 0.773 |  | -0.0122 | 0.023 | -0.521 | 0.604 |  | 0.0213 | 0.016 | 1.345 | 0.182 |  | 0.0134 | 0.017 | 0.803 | 0.424 |  |
| BMI | -0.0032 | 0.017 | -0.188 | 0.851 |  | -0.0342 | 0.019 | -1.847 | 0.068 | . | -0.0268 | 0.023 | -1.157 | 0.250 |  | 0.0123 | 0.020 | 0.630 | 0.530 |  | -0.0130 | 0.016 | -0.790 | 0.432 |  | 0.0202 | 0.019 | 1.059 | 0.292 |  | -0.0057 | 0.013 | -0.445 | 0.657 |  | -0.0003 | 0.013 | -0.024 | 0.981 |  |
| HbA1c | -0.0102 | 0.018 | -0.570 | 0.570 |  | 0.0143 | 0.019 | 0.747 | 0.457 |  | -0.0057 | 0.024 | -0.238 | 0.813 |  | -0.0172 | 0.020 | -0.848 | 0.399 |  | 0.0155 | 0.017 | 0.900 | 0.370 |  | 0.0238 | 0.020 | 1.194 | 0.235 |  | 0.0247 | 0.013 | 1.863 | 0.066 | . | 0.0086 | 0.014 | 0.618 | 0.538 |  |
| WMH | 0.0056 | 0.023 | 0.237 | 0.813 |  | 0.0145 | 0.025 | 0.575 | 0.567 |  | 0.0135 | 0.032 | 0.428 | 0.670 |  | 0.0247 | 0.027 | 0.924 | 0.358 |  | 0.0262 | 0.022 | 1.185 | 0.240 |  | 0.0039 | 0.026 | 0.153 | 0.879 |  | -0.0052 | 0.017 | -0.302 | 0.763 |  | 0.0085 | 0.018 | 0.467 | 0.642 |  |
| Aβ | 0.0289 | 0.020 | 1.462 | 0.148 |  | 0.0190 | 0.021 | 0.891 | 0.375 |  | 0.0489 | 0.027 | 1.834 | 0.070 | . | 0.0422 | 0.022 | 1.877 | 0.064 | . | -0.0073 | 0.019 | -0.388 | 0.699 |  | -0.0031 | 0.022 | -0.145 | 0.885 |  | 0.0048 | 0.015 | 0.329 | 0.743 |  | 0.0100 | 0.015 | 0.648 | 0.519 |  |
| Age | -0.0233 | 0.027 | -0.850 | 0.398 |  | -0.0168 | 0.030 | -0.571 | 0.570 |  | -0.0388 | 0.037 | -1.050 | 0.297 |  | -0.0457 | 0.031 | -1.470 | 0.145 |  | -0.0091 | 0.026 | -0.352 | 0.726 |  | -0.0364 | 0.030 | -1.219 | 0.226 |  | 0.0227 | 0.020 | 1.119 | 0.266 |  | -0.0073 | 0.021 | -0.341 | 0.734 |  |
| sex (women to men) | -0.0403 | 0.034 | -1.198 | 0.234 |  | -0.0023 | 0.036 | -0.062 | 0.950 |  | -0.0348 | 0.045 | -0.766 | 0.446 |  | -0.0521 | 0.038 | -1.358 | 0.178 |  | -0.0731 | 0.032 | -2.298 | 0.024 | * | -0.1037 | 0.037 | -2.826 | 0.006 | ** | -0.0437 | 0.025 | -1.758 | 0.082 | . | -0.0437 | 0.026 | -1.669 | 0.099 | . |
| GM volume | -0.0085 | 0.025 | -0.340 | 0.734 |  | 0.0122 | 0.026 | 0.461 | 0.645 |  | 0.0015 | 0.033 | 0.046 | 0.963 |  | 0.0077 | 0.028 | 0.277 | 0.783 |  | 0.0291 | 0.024 | 1.194 | 0.235 |  | -0.0165 | 0.028 | -0.589 | 0.557 |  | 0.0133 | 0.019 | 0.700 | 0.485 |  | 0.0061 | 0.020 | 0.308 | 0.759 |  |
| mean FD | -0.0934 | 0.016 | -5.894 | 0.000 | *** | -0.0796 | 0.016 | -4.932 | 0.000 | *** | -0.1142 | 0.019 | -5.861 | 0.000 | *** | -0.1117 | 0.017 | -6.536 | 0.000 | *** | -0.0735 | 0.016 | -4.469 | 0.000 | *** | -0.0930 | 0.019 | -4.860 | 0.000 | *** | -0.0448 | 0.013 | -3.572 | 0.000 | *** | -0.0847 | 0.013 | -6.563 | 0.000 | *** |
| diagnostic (SCD to OA) | 0.0314 | 0.046 | 0.687 | 0.494 |  | 0.1154 | 0.049 | 2.333 | 0.022 | * | 0.0311 | 0.062 | 0.502 | 0.617 |  | 0.1216 | 0.052 | 2.332 | 0.022 | * | -0.0483 | 0.043 | -1.123 | 0.265 |  | -0.0215 | 0.049 | -0.435 | 0.665 |  | -0.0131 | 0.034 | -0.389 | 0.698 |  | 0.0061 | 0.035 | 0.173 | 0.863 |  |
| diagnostic (MCI to OA) | 0.0249 | 0.040 | 0.621 | 0.536 |  | 0.0359 | 0.043 | 0.833 | 0.407 |  | 0.0095 | 0.054 | 0.176 | 0.860 |  | -0.0091 | 0.045 | -0.201 | 0.841 |  | 0.0169 | 0.038 | 0.447 | 0.656 |  | -0.0156 | 0.043 | -0.358 | 0.721 |  | -0.0190 | 0.030 | -0.643 | 0.522 |  | 0.0110 | 0.031 | 0.353 | 0.725 |  |
|  |  |  |  |  |  |  |  |  |  |  |  |  |  |  |  |  |  |  |  |  |  |  |  |  |  |  |  |  |  |  |  |  |  |  |  |  |  |  |  |  |
| R2m | 0.246 |  |  |  |  | 0.274 |  |  |  |  | 0.277 |  |  |  |  | 0.311 |  |  |  |  | 0.170 |  |  |  |  | 0.229 |  |  |  |  | 0.161 |  |  |  |  | 0.250 |  |  |  |  |
| R2c | 0.544 |  |  |  |  | 0.624 |  |  |  |  | 0.679 |  |  |  |  | 0.617 |  |  |  |  | 0.344 |  |  |  |  | 0.274 |  |  |  |  | 0.424 |  |  |  |  | 0.522 |  |  |  |  |

Using linear mixed-effects models, we assessed resting-state functional connectivity (RSFC) trajectories across the entire study sample within seven functional networks as defined by the Schaefer parcellation atlas (DMN, default mode network; SAL/VAN, salience and ventral attention network; FPN, fronto-parietal network; LIM, limbic network; DAN, dorsal attention network; VIS, visual network and SM, somatomotor network) and throughout the whole brain (global). We investigated the associations between vascular risk factors (VRF; systolic blood pressure, sBP; diastolic blood pressure, dBP; body-mass-index, BMI; glycated hemoglobin A1, HbA1c), white matter hyperintensities (WMH), and Amyloid-β (Aβ) and longitudinal changes in RSFC over time. A random effects intercept and slope of RSFC for each individual were included in the models. Models were corrected for baseline age, sex, gray matter volume (GMV), diagnostic group, for longitudinal mean frame-displacement (FD) and for the interactions of selected covariates with time. Unstandardized estimates (Est.), standard errors (SE), T values and P values (****p* < 0.001, ***p* < 0.01, **p* < 0.05) as well as marginal (R2m) and conditional (R2c) R^2^ values are presented. *Other abbreviations:* Obs., observations; part, participants.

## Table S2. Association between longitudinal changes in RSFC and changes in cognitive performance

|  | **Executive Function** | | | |  | **Episodic Memory** | | | |  | **Processing Speed** | | | |  | **Working Memory** | | | |  |
| --- | --- | --- | --- | --- | --- | --- | --- | --- | --- | --- | --- | --- | --- | --- | --- | --- | --- | --- | --- | --- |
|  | n of obs. = 223 | | n of part. = 88 | |  | n of obs. = 235 | | n of part. = 92 | |  | n of obs. = 230 | | n of part. = 90 | |  | n of obs. = 242 | | n of part. = 94 | |  |
|  |  |  |  |  |  |  |  |  |  |  |  |  |  |  |  |  |  |  |  |  |
|  | **Est.** | **SE** | **t** | **p** |  | **Est.** | **SE** | **t** | **p** |  | **Est.** | **SE** | **t** | **p** |  | **Est.** | **SE** | **t** | **p** |  |
| DMN slope × time | -0.1320 | 0.241 | -0.547 | 0.587 |  | 0.0093 | 0.033 | 0.279 | 0.781 |  | -0.0049 | 0.059 | -0.082 | 0.935 |  | -0.0064 | 0.030 | -0.209 | 0.835 |  |
| age × time | 0.0356 | 0.337 | 0.105 | 0.916 |  | -0.1425 | 0.044 | -3.214 | 0.002 | ** | -0.0882 | 0.082 | -1.078 | 0.284 |  | -0.0941 | 0.041 | -2.290 | 0.024 | * |
| sex (women to men) × time | -0.4761 | 0.517 | -0.921 | 0.361 |  | -0.0255 | 0.070 | -0.366 | 0.715 |  | -0.0629 | 0.124 | -0.505 | 0.615 |  | -0.0088 | 0.064 | -0.137 | 0.892 |  |
| education × time | -0.0643 | 0.253 | -0.254 | 0.800 |  | 0.0102 | 0.033 | 0.305 | 0.761 |  | 0.0826 | 0.060 | 1.370 | 0.174 |  | -0.0124 | 0.030 | -0.409 | 0.683 |  |
| DMN slope | -0.1516 | 0.201 | -0.756 | 0.452 |  | -0.0127 | 0.140 | -0.090 | 0.928 |  | -0.0182 | 0.102 | -0.179 | 0.858 |  | -0.0472 | 0.073 | -0.644 | 0.521 |  |
| time (cognition) | -0.0091 | 0.359 | -0.025 | 0.980 |  | 0.0502 | 0.049 | 1.019 | 0.312 |  | -0.0303 | 0.087 | -0.348 | 0.729 |  | 0.0245 | 0.045 | 0.543 | 0.589 |  |
| age | -0.2308 | 0.264 | -0.873 | 0.385 |  | -0.4254 | 0.172 | -2.475 | 0.015 | * | -0.4468 | 0.130 | -3.445 | 0.001 | *** | -0.2023 | 0.090 | -2.250 | 0.027 | * |
| sex (women to men) | -0.3403 | 0.419 | -0.812 | 0.419 |  | 0.5212 | 0.282 | 1.851 | 0.068 | . | 0.1374 | 0.206 | 0.668 | 0.506 |  | -0.1270 | 0.148 | -0.856 | 0.395 |  |
| education | -0.1892 | 0.206 | -0.920 | 0.361 |  | 0.3004 | 0.135 | 2.223 | 0.029 | * | 0.2331 | 0.100 | 2.331 | 0.022 | * | 0.2443 | 0.071 | 3.418 | 0.001 | *** |
|  |  |  |  |  |  |  |  |  |  |  |  |  |  |  |  |  |  |  |  |  |
| R2m | 0.026 |  |  |  |  | 0.134 |  |  |  |  | 0.137 |  |  |  |  | 0.143 |  |  |  |  |
| R2c | 0.282 |  |  |  |  | 0.937 |  |  |  |  | 0.788 |  |  |  |  | 0.768 |  |  |  |  |
|  |  |  |  |  |  |  |  |  |  |  |  |  |  |  |  |  |  |  |  |  |
|  | **Est.** | **SE** | **t** | **p** |  | **Est.** | **SE** | **t** | **p** |  | **Est.** | **SE** | **t** | **p** |  | **Est.** | **SE** | **t** | **p** |  |
| SAL/VAN slope × time | -0.1331 | 0.240 | -0.555 | 0.581 |  | 0.0229 | 0.033 | 0.695 | 0.490 |  | -0.0161 | 0.059 | -0.271 | 0.787 |  | 0.0031 | 0.030 | 0.103 | 0.918 |  |
| age × time | 0.0395 | 0.337 | 0.117 | 0.907 |  | -0.1435 | 0.044 | -3.247 | 0.002 | ** | -0.0887 | 0.082 | -1.085 | 0.281 |  | -0.0939 | 0.041 | -2.283 | 0.025 | * |
| sex (women to men) × time | -0.4739 | 0.516 | -0.918 | 0.362 |  | -0.0263 | 0.069 | -0.379 | 0.706 |  | -0.0620 | 0.124 | -0.499 | 0.619 |  | -0.0120 | 0.064 | -0.187 | 0.852 |  |
| education × time | -0.0768 | 0.252 | -0.305 | 0.762 |  | 0.0109 | 0.033 | 0.328 | 0.744 |  | 0.0818 | 0.060 | 1.358 | 0.178 |  | -0.0135 | 0.030 | -0.447 | 0.656 |  |
| SAL/VAN slope | -0.2197 | 0.201 | -1.095 | 0.277 |  | -0.0466 | 0.141 | -0.330 | 0.742 |  | -0.0557 | 0.102 | -0.547 | 0.586 |  | 0.0278 | 0.074 | 0.376 | 0.708 |  |
| time (cognition) | -0.0131 | 0.359 | -0.037 | 0.971 |  | 0.0498 | 0.049 | 1.013 | 0.315 |  | -0.0308 | 0.087 | -0.353 | 0.725 |  | 0.0253 | 0.045 | 0.560 | 0.577 |  |
| age | -0.2321 | 0.264 | -0.880 | 0.381 |  | -0.4260 | 0.172 | -2.481 | 0.015 | * | -0.4491 | 0.129 | -3.469 | 0.001 | *** | -0.2021 | 0.090 | -2.242 | 0.027 | * |
| sex (women to men) | -0.3486 | 0.418 | -0.834 | 0.407 |  | 0.5182 | 0.281 | 1.841 | 0.069 | . | 0.1365 | 0.205 | 0.666 | 0.508 |  | -0.1282 | 0.149 | -0.862 | 0.391 |  |
| education | -0.2065 | 0.205 | -1.006 | 0.317 |  | 0.2979 | 0.135 | 2.202 | 0.030 | * | 0.2294 | 0.100 | 2.294 | 0.024 | * | 0.2443 | 0.072 | 3.406 | 0.001 | *** |
|  |  |  |  |  |  |  |  |  |  |  |  |  |  |  |  |  |  |  |  |  |
| R2m | 0.029 |  |  |  |  | 0.135 |  |  |  |  | 0.139 |  |  |  |  | 0.141 |  |  |  |  |
| R2c | 0.283 |  |  |  |  | 0.937 |  |  |  |  | 0.788 |  |  |  |  | 0.769 |  |  |  |  |
|  |  |  |  |  |  |  |  |  |  |  |  |  |  |  |  |  |  |  |  |  |
|  | **Est.** | **SE** | **t** | **p** |  | **Est.** | **SE** | **t** | **p** |  | **Est.** | **SE** | **t** | **p** |  | **Est.** | **SE** | **t** | **p** |  |
| FPN slope × time | -0.2894 | 0.240 | -1.207 | 0.233 |  | -0.0067 | 0.033 | -0.204 | 0.839 |  | -0.0133 | 0.059 | -0.226 | 0.822 |  | -0.0010 | 0.030 | -0.034 | 0.973 |  |
| age × time | 0.0275 | 0.335 | 0.082 | 0.935 |  | -0.1433 | 0.044 | -3.233 | 0.002 | ** | -0.0887 | 0.082 | -1.085 | 0.281 |  | -0.0939 | 0.041 | -2.285 | 0.025 | * |
| sex (women to men) × time | -0.4740 | 0.512 | -0.926 | 0.358 |  | -0.0227 | 0.070 | -0.326 | 0.745 |  | -0.0619 | 0.124 | -0.498 | 0.620 |  | -0.0094 | 0.064 | -0.147 | 0.884 |  |
| education × time | -0.0572 | 0.251 | -0.228 | 0.820 |  | 0.0111 | 0.033 | 0.332 | 0.741 |  | 0.0832 | 0.060 | 1.380 | 0.171 |  | -0.0126 | 0.030 | -0.416 | 0.679 |  |
| FPN slope | -0.0572 | 0.202 | -0.283 | 0.778 |  | -0.0084 | 0.139 | -0.060 | 0.952 |  | -0.0869 | 0.101 | -0.861 | 0.392 |  | -0.0635 | 0.073 | -0.864 | 0.390 |  |
| time (cognition) | -0.0083 | 0.356 | -0.023 | 0.981 |  | 0.0491 | 0.049 | 0.996 | 0.323 |  | -0.0306 | 0.087 | -0.351 | 0.727 |  | 0.0250 | 0.045 | 0.554 | 0.581 |  |
| age | -0.2336 | 0.265 | -0.881 | 0.380 |  | -0.4253 | 0.172 | -2.475 | 0.015 | * | -0.4494 | 0.129 | -3.482 | 0.001 | *** | -0.2015 | 0.090 | -2.245 | 0.027 | * |
| sex (women to men) | -0.3388 | 0.420 | -0.806 | 0.422 |  | 0.5219 | 0.282 | 1.853 | 0.067 | . | 0.1358 | 0.205 | 0.663 | 0.509 |  | -0.1279 | 0.148 | -0.864 | 0.390 |  |
| education | -0.1856 | 0.206 | -0.899 | 0.371 |  | 0.2997 | 0.135 | 2.218 | 0.029 | * | 0.2339 | 0.100 | 2.350 | 0.021 | * | 0.2440 | 0.071 | 3.420 | 0.001 | *** |
|  |  |  |  |  |  |  |  |  |  |  |  |  |  |  |  |  |  |  |  |  |
| R2m | 0.032 |  |  |  |  | 0.134 |  |  |  |  | 0.140 |  |  |  |  | 0.145 |  |  |  |  |
| R2c | 0.281 |  |  |  |  | 0.937 |  |  |  |  | 0.787 |  |  |  |  | 0.767 |  |  |  |  |

Using linear mixed-effects models (LME), we explored the associations between the VRF-related changes in resting-state functional connectivity (RSFC) and longitudinal changes in cognitive performance. The RSFC slopes of the default mode network (DMN), the salience and ventral attention network (SAL/VAN), and the fronto-parietal network (FPN) were derived from the significant predicted LMEs. Cognitive performance was represented by four cognitive composite scores, i.e. executive functions, episodic memory, processing speed and working memory. As random effects intercept and slope of cognitive performance for each individual were included in the models. Models were corrected for baseline age, sex and education and for the interactions of those covariates with time. Unstandardized estimates (Est.), standard errors (SE), T values and P values (****p* < 0.001, ***p* < 0.01, **p* < 0.05) as well as marginal (R2m) and conditional (R2c) R2 values are presented. *Abbreviations:* VRF, vascular risk factor; Obs., observations; part, participants.
